# Can Grapevine Leaf Water Potential Be Modelled from Physiological and Meteorological Variables? A Machine Learning Approach

**DOI:** 10.3390/plants12244142

**Published:** 2023-12-12

**Authors:** Miguel Damásio, Miguel Barbosa, João Deus, Eduardo Fernandes, André Leitão, Luís Albino, Filipe Fonseca, José Silvestre

**Affiliations:** 1 INIAV I.P., Instituto Nacional de Investigação Agrária e Veterinária, Polo de Inovação de Dois Portos, Quinta da Almoinha, 2565-191 Dois Portos, Portugal; joao.deus@iniav.pt (J.D.); jose.silvestre@iniav.pt (J.S.); 2 SISCOG SA, Sistemas Cognitivos, Campo Grande, 378 - 3°, 1700-097 Lisboa, Portugal; miguel.barbosa@siscog.pt (M.B.); eduardo.fernandes@siscog.pt (E.F.); andre.leitao@siscog.pt (A.L.); luis.albino@siscog.pt (L.A.); filipe.fonseca@siscog.pt (F.F.); 3 GREEN-IT Bioresources4sustainability, ITQB NOVA, Av. da República, 2780-157 Oeiras, Portugal; 4 GI-1716 Projects and Planification, Agroforestry Engineering Department, Escuela Politécnica Superior de Ingeniería Lugo, University of Santiago de Compostela, 27002 Lugo, Spain

**Keywords:** *Vitis vinifera*, water status indicators, modelling, predawn leaf water potential, precision irrigation

## Abstract

Climate change is affecting global viticulture, increasing heatwaves and drought. Precision irrigation, supported by robust water status indicators (WSIs), is inevitable in most of the Mediterranean basin. One of the most reliable WSIs is the leaf water potential ( $\Psi_{leaf}$ ), which is determined via an intrusive and time-consuming method. The aim of this work is to discern the most effective variables that are correlated with plants’ water status and identify the variables that better predict $\Psi_{leaf}$ . Five grapevine varieties grown in the Alentejo region (Portugal) were selected and subjected to three irrigation treatments, starting in 2018: full irrigation (FI), deficit irrigation (DI), and no irrigation (NI). Plant monitoring was performed in 2023. Measurements included stomatal conductance ( gs ), predawn water potential $\Psi_{pd}$ , stem water potential ( $\Psi_{stem}$ ), thermal imaging, and meteorological data. The WSIs, namely $\Psi_{pd}$ and gs , responded differently according to the irrigation treatment. $\Psi_{stem}$ measured at mid-morning (MM) and mid-day (MD) proved unable to discern between treatments. MM measurements presented the best correlations between WSIs. gs showed the best correlations between the other WSIs, and consequently the best predictive capability to estimate $\Psi_{pd}$ . Machine learning regression models were trained on meteorological, thermal, and gs data to predict $\Psi_{pd}$ , with ensemble models showing a great performance (ExtraTrees: $R^{2}=0.833$ , MAE=0.072 ; Gradient Boosting: $R^{2}=0.830$ ; MAE=0.073 ).

## 1. Introduction

The Mediterranean region is profoundly influenced by grape and wine production. The viticulture and wine sectors are important components of Mediterranean economies [1]. In 2022, it was estimated that the world wine production ranged between 257.5 and 263.3 million hl, with the three largest producers being European Mediterranean countries—Italy, France, and Spain [2]. Looking more closely at Portugal, it stood as the 10th and 11th biggest global wine producer and consumer, respectively [2].

Climate change stands as a pivotal environmental concern of the 21st century, as underscored by the Intergovernmental Panel on Climate Change [3]. According to the same reference, global temperatures will increase by 1.4 °C (RCP (representative concentration pathway) 2.6), 2.7 °C (RCP 4.5), or 4.4 °C (RCP 8.5) by 2081–2100. Projections for the Mediterranean basin [4] point to an increase of 0.5 to 2.5 °C (RCP 2.6) or 3.8 to 6.5 °C (RCP 8.5) by 2100. Given the Mediterranean-like climate of mainland Portugal—characterised by warm, dry summers and mild to wet winters—these climatic alterations will exacerbate aridity and precipitation patterns [5,6]. *Vitis vinifera* L., the grapevine species, is acknowledged for being a “drought-tolerant” species, finding suitability in semi-arid regions with Mediterranean climate characteristics typified by warm, arid summers, although this classification is not agreed [7,8]. Water scarcity, particularly water stress and even drought, emerges as the foremost challenge for the viticulture industry [9,10,11]. Notably, extreme events, exemplified by heat waves, imperil productivity and quality. These events can trigger wide-scale grape berry sunburn and defoliation due to elevated temperatures and severe water stress, culminating in source/sink ratio imbalances, reduced photosynthetic rates, and a compromised carbon uptake. Furthermore, these events may hinder proper grape maturation, influencing the overall winemaking process [12,13,14]. The urgency lies in deciphering the mechanisms underlying grapevine adaptation in the face of these evolving conditions to facilitate the implementation of short- and long-term mitigation strategies.

Deficit irrigation has emerged as a prospective strategy to bolster the resilience of crops in the face of mild to severe water stress, as documented by Costa et al. [15], Zarrouk et al. [16], and Galindo et al. [17], among others. In the context of wine growing, enhancing the efficiency of water utilisation through judicious deficit irrigation management assumes paramount significance, due to the effect of the grapevine water status on wine quality [18,19,20]. This necessitates the adoption of cultural practices that facilitate the employment of irrigation water, exemplified by strategies like regulated deficit irrigation (RDI) [14,16]. These practices constitute indispensable pillars for establishing a sustainable and viable future for viticulture.

Critical to the evolution of viticultural practices is the comprehensive examination of grape varieties’ adaptability to scenarios characterised by extreme water deficits. This necessity originates from the potential for winegrowers to proactively select grape varieties and rootstocks that align with the anticipated climate patterns in their respective regions. The rootstocks’ genetic variability can be used by winegrowers, considering that different rootstocks confer different levels of tolerance to drought. Drought-tolerant rootstocks allow for a good yield and vegetative growth under water stress conditions [21]. It is important to underscore that the degree of adaptability is inherently influenced by the specific genotype employed [11,22,23]. To optimise deficit irrigation management and ascertain the most compatible grape varieties, it is important to unravel the physiological and molecular foundations governing water dynamics and movement at both the cellular and tissue levels. This endeavour extends to understanding these processes within the entirety of grapevine organisms, encompassing diverse genotypic variations. Consequently, unravelling the intricate mechanisms of water relations and movement within grapevines remains an essential frontier in the endeavour to elevate viticultural practices [16,24].

The interplay between productivity, plant survival, and physiological processes is contingent upon the intricate dynamics of carbon dioxide (CO_2_) uptake through stomatal regulation and photosynthetic carbon fixation at the leaf level [25,26,27]. This interdependence is entwined with the plants’ water status and the equilibrium of water within leaves. The context of this equilibrium gains prominence due to the influence of evaporative demand, characterised by a heightened vapour pressure deficit (VPD), which, when coupled with the leaf’s water deficit, can elicit stomatal closure [28,29]. Consequently, this closure leads to constraints in photosynthetic rates, aligning with observations made by Sperry [30] and Trifiló et al. [31]. This closure of stomata is quantified as stomatal conductance ( gs ), and concerns CO_2_ and water vapour exchange. The pivotal role of stomata extends to the regulation of both the transpiration rate (E), which has implications for leaf temperature modulation, and the uptake of CO_2_ [13]. Under conditions of a water deficit, stomatal closure emerges as a critical mechanism that not only curtails photosynthesis, as elucidated by Flexas and Medrano [13], but also exerts constraints on temperature moderation and may precipitate leaf senescence [32]. Stomatal behaviour exhibits temporal dynamics, with the potential for complete aperture during periods of low light intensity and elevated air humidity in the morning or evenings, a state accompanied by subdued photosynthetic rates and, consequently, a diminished water use efficiency (WUE). This openness contrasts with the mid-day (MD) response to an elevated VPD, where stomata tend to close to counteract water loss, elevating the WUE if photosynthesis is sustained, an adaptation detailed by Souza et al. [33] and Chaves et al. [14]. This orchestrated response to a heightened VPD underscores its role as an indispensable mechanism in water conservation strategies.

The water demands of crops are subject to the interplay of two fundamental determinants: inherent attributes of the crop itself, encompassing its vitality, and environmental factors related to the soil total available water and evapotranspiration (ET) rates. In conjunction with meteorological data, the integration of remote sensing (RS) technology permits farmers to acquire an understanding of spatial discrepancies within cultivation plots. Consequently, this knowledge empowers them to apply differential irrigation management, optimising the efficiency of water use [34]. In contemporary times, numerous investigations have established associations between RS-derived vegetation indices (VIs) and crop coefficients for an extensive spectrum of crops. These correlations have been documented in studies by Bausch et al. [35], Choudhury et al. [36], Hunsaker et al. [37] and Johnson et al. [38], encompassing both perennial and enduring crop varieties [39,40,41,42]. Various research endeavours have further leveraged these VIs to appraise the requisite water volumes for crops [43,44,45,46,47,48]. Such insights serve as valuable tools for aligning irrigation practices with the authentic water requisites of crops. RS-based methodologies also harbour substantial potential in gauging the severity of plant water stress, as exemplified by metrics like the Crop Water Stress Index (CWSI) [49] or the Shortwave Infrared Water Stress Index (SIWSI) [50]. Alternate RS strategies have yielded frameworks such as the Surface Energy Balance Algorithm for Land (SEBAL) [51] or Mapping ET at High Resolution with Internalised Calibration (METRIC) [52]. These approaches facilitate the estimation of the sensible heat flux (H), a pivotal parameter for the precise evaluation of ongoing crop ET rates. Conversely, the amalgamation of thermal imagery featuring an elevated spatial resolution with on-site meteorological data holds promise in the estimation of distinct ET components, encompassing evaporation and E.

The trade-off between quantity and quality in winemaking is a well-known relation to farmers that requires fine-tuning the vine balance to achieve production goals. This can only be achieved through having a robust estimate of the plant response to water inputs.

A general but controversial classification of varieties [53] regarding the stomatal response to water stress is the iso/anisohydric terminology, introduced by Schultz [54]. Generally speaking, isohydric varieties tend to be more responsive to water deficits, closing the stomata earlier and presenting a lower variation in leaf water potential ( $\Psi_{leaf}$ ) in relation to anisohydric varieties that, under the same water deficit conditions, maintain a higher E and lower $\Psi_{leaf}$ [55]. The varieties’ iso/anisohydric behaviour depends on the interaction of many characteristics, such as rootstock, meteorological conditions, and the intensity and duration of water deficits [56].

$\Psi_{leaf}$ measurements have been largely used as a way to survey grapevines’ water status [57,58]. Despite being correlated with many parameters of grapevine physiology, vegetative growth, reproductive growth, and yield, it has been observed that these correlations are not as strong during MD or diurnal measurements [58]. The predawn leaf water potential ( $\Psi_{pd}$ ) and the stem water potential ( $\Psi_{stem}$ ) seem to enable more accurate estimations of the grapevine water status. The first is measured before sunrise when it is expected that the leaves are in equilibrium with the soil’s water potential, and has proven to be more reliable than $\Psi_{leaf}$ for estimating water stress [59]. $\Psi_{stem}$ is measured by enclosing a leaf in a plastic bag wrapped in aluminium foil and has been found to be accurate in estimating water status differences between treatments [60]. However, there are limitations to these methodologies. As Améglio et al. [61] concluded, in a work with potted and soil-planted walnut trees, $\Psi_{pd}$ is a good indicator for water stress in most cases, but not so much for crops under conditions of heterogeneous soil humidity. A small humid soil layer might be able to rehydrate a vine during the night, bringing $\Psi_{pd}$ to values close to zero, but might not be able to provide enough water to the vine during the day to meet the evaporative demand. In these cases, other approaches, which are not biased by the spatial distribution of soil water, can complement $\Psi_{pd}$ measurements. Furthermore, the VPD and night-time E can affect $\Psi_{pd}$ [62]. According to van Leeuwen et al. [18], $\Psi_{stem}$ is a useful tool to manage irrigation since it accurately describes the whole-vine water status. However, it must be noted that this measurement is also affected by meteorological conditions, and the threshold for permanent damage to the canopy and grapes varies with the vines’ vigour. For example, van Leeuwen et al. [18] report that in vigorous vines, embolism may occur at −1.2 megapascal (MPa), while the same damage occurs at −1.6 MPa on low-vigour vines that are progressively exposed to water deficits. Furthermore, the species and varieties’ isohydric or anisohydric behaviour is critical when interpreting $\Psi_{stem}$ . Species with isohydric behaviour present a high degree of stomatal control, maintaining their $\Psi_{stem}$ at a relatively constant value, and may not accurately reflect the severity of water deficits. On the other hand, species with anisohydric behaviour present more dynamic changes in $\Psi_{stem}$ in response to variations in soil moisture, potentially overestimating the severity of water deficits [63]. These dynamics may reduce $\Psi_{stem}$ ’s usefulness as a water status indicator (WSI). Moreover, water potential measurements are laborious, invasive, and time-consuming and require specific instruments that are difficult to transport (gas canisters, Scholander pressure chambers), unlike other measurements like gas exchange or thermal imaging.

gs is one of the most reliable WSIs [64] and can provide additional information on how the vine is responding to its water status from a physiological point of view [65] with well-defined thresholds for distinct levels of water stress [66]. Other reliable WSIs are derived from the canopy temperature. In fact, the canopy temperature has been proposed for many years as a tool to determine the plants’ water status [67]. Thermal sensing has been shown to be a valuable tool for estimating a plant’s water status in viticulture [68,69,70]. As technology progresses, the cost of thermal cameras has become accessible for these kinds of estimations by farmers and technicians, making access to the data an easy task [71]. The major bottleneck is the subsequent data treatment, which, depending on the dataset volume, can be an extremely time-consuming endeavour. It must be said, however, that the accuracy of these thermal indicators can be reduced by adverse weather conditions [72].

It is well known that, in recent years, machine learning techniques have had a large impact on the development of agricultural decision-support systems by making use of multisource data to fit models that provide agricultural insights and estimates, classify valuable indicators of plant status, and predict crop outputs. Some examples are plant breeding [73], in vitro culture [74], stress phenotyping [75], stress physiology [76], plant system biology [77], plant identification [78], plant genetic engineering [79], and pathogen identification [80]. A major revision that encompasses general machine learning techniques and their application in the field of agriculture was published by Benos et al. [81].

Specifically, in the domain of crop water management, efficient estimation of the plants’ water status can provide the farmer with a quick and reliable tool for aiding irrigation management. Water potential ( Ψ ) estimation with machine learning methods offers a non-invasive and efficient method of evaluating the plants’ water status. The current literature on the estimation of $\Psi_{stem}$ or $\Psi_{leaf}$ in vineyards generally focuses on the creation of indices derived from multispectral [82,83,84,85,86,87,88] or hyperspectral data [89,90,91,92,93,94,95,96]. Thermal indices, most commonly the CWSI, show a good correlation with the MD $\Psi_{leaf}$ [97,98], whereas VIs used alone [83,87] or in combination with thermal indices have also shown acceptable results [95].

In conclusion, with this work, we intended to (i) evaluate which of the two Ψ methods ( $\Psi_{pd}$ and $\Psi_{stem}$ ) is more robust in determining the plants’ water status in an arid region with high evaporative demand. (ii) Identify the variables that demonstrate superior predictive capabilities in forecasting the more robust Ψ method. As Ψ is generally used by farmers as one of the main WSIs for irrigation scheduling, the accomplishment of these objectives will allow for a better management of RDI by farmers. Finally, (iii) model the Ψ selected in point (i). To achieve this, an array of mathematical models was meticulously evaluated and scrutinised to determine their efficacy in capturing the intricate relationships under investigation. This work provides the foundation for mapping out water stress across a vineyard by accurately estimating Ψ at the plant level from sparse observations that can be used as anchor points for extrapolating information collected from other platforms. Methods of estimation range from simple linear regression (SLR) [82,83,85,86,89,94,95,97] and partial least squares (PLS) [88,91,96] to ensemble models [90,91,93,95] and neural networks [84,87]. When employing machine learning methods, input data typically assume indices obtained from spectral data [87,97,98] or the raw spectral bands [89,90,92] to determine the best spectral domains for the plant water assessment. Meteorology data [88] or others, such as soil data [99], might also provide useful inputs to the modelling algorithms. This investigation encompassed five distinct grapevine varieties, systematically chosen based on their contrasting carbon isotopic signatures [27].

## 2. Results

This work was performed from day of the year (DOY) 177 to 181 and from DOY 193 to 195, and partially coincided with the Canadian wild fires, which produced a cloud of smoke that traversed the skies starting at DOY 178 and dissipated at DOY 180 [100]. While the effects were not significant at DOY 178, the same cannot be said of DOY 180. This event clearly affected the thermographic and gs values at DOY 180.

### 2.1. Correlations between WSI

Pearson Product–Moment Correlation Coefficients between the WSIs (CWSI, gs , $\Psi_{pd}$ , $\Psi_{stem}$ and the normalised canopy temperature ( ΔT )) were determined for each variety and each measurement period: mid-morning (MM) and MD.

**Figure 1:**
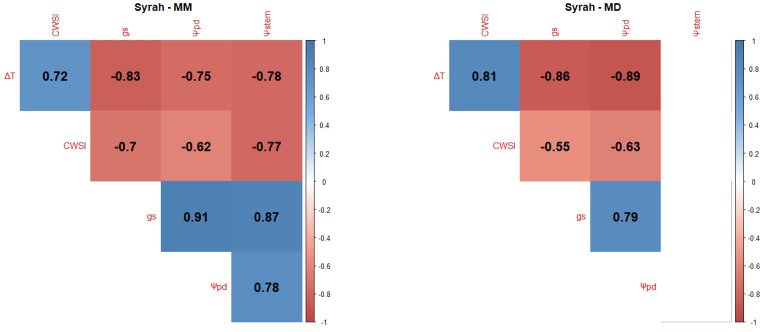
Correlograms showing Pearson Product–Moment Correlation Coefficients of the variety Sy. Non-significant correlation coefficients (*p* > 0.05) are displayed in white with no value. Positive correlations are represented in blue and negative correlations are represented in red. The variety’s name is followed by MM for the correlation between MM measurements (**left side**) or MD for the correlation between mid-day measurements (**right side**).

Syrah (Sy) presented a high degree of correlation between all variables (Figure 1), especially in the MM measurements. There are strong positive correlations between $\Psi_{pd}$ with ΔT , and strong negative correlations of ΔT with gs in the MM measurement, and correlations of ΔT with $\Psi_{pd}$ , gs , and the CWSI (negative, negative, and positive correlations, respectively) in the MD measurement. During the latter measurement, there were no correlations between $\Psi_{stem}$ and all the other variables.

**Figure 2:**
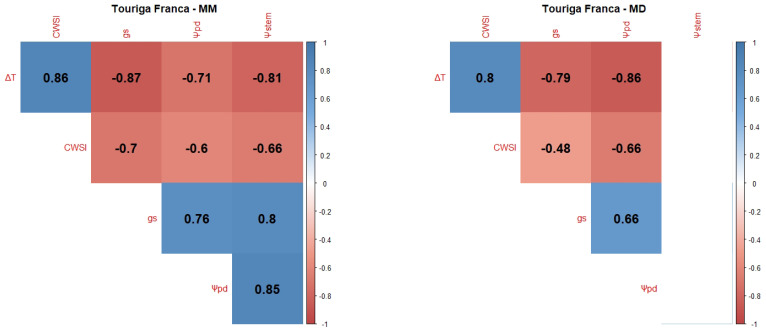
Correlograms showing Pearson Product–Moment Correlation Coefficients of the variety TF. Non-significant correlation coefficients (*p* > 0.05) are displayed in white with no value. Positive correlations are represented in blue and negative correlations are represented in red. The variety’s name is followed by MM for the correlation between MM measurements (**left side**) or MD for the correlation between mid-day measurements (**right side**).

Touriga Franca (TF) presented strong correlations between ΔT and the other variables (Figure 2); in particular, there was a negative correlation with gs and a positive correlation with the CWSI in the MM measurement, and a negative correlation with $\Psi_{pd}$ and a positive correlation with the CWSI in the MD measurement. In the MM measurement, there was also a strong correlation between $\Psi_{pd}$ and $\Psi_{stem}$ . The MD correlations are not as strong as the MM ones, and, like Sy, $\Psi_{stem}$ presented no correlations with the other variables.

**Figure 3:**
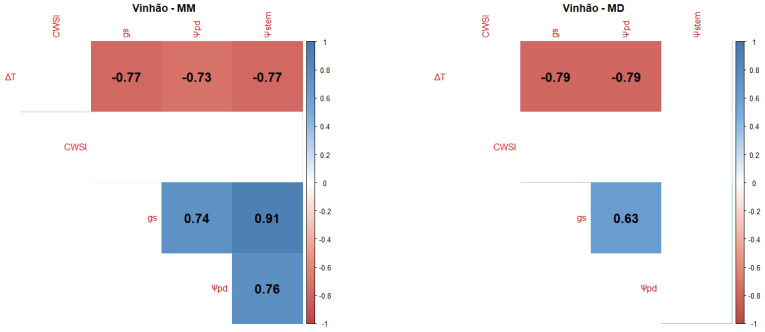
Correlograms showing Pearson Product–Moment Correlation Coefficients of the variety Vi. Non-significant correlation coefficients (*p* > 0.05) are displayed in white with no value. Positive correlations are represented in blue and negative correlations are represented in red. The variety’s name is followed by MM for the correlation between MM measurements (**left side**) or MD for the correlation between mid-day measurements (**right side**).

Vinhão (Vi) was by far the variety that presented the worst correlations (Figure 3), especially during the MD measurements. The best correlations were achieved between gs and the other variables, especially $\Psi_{pd}$ (positive correlation) in the MM measurement. On the other hand, the CWSI presented no correlations with the other variables in the two measurements, and $\Psi_{stem}$ presented no correlations in the MD measurement.

**Figure 4:**
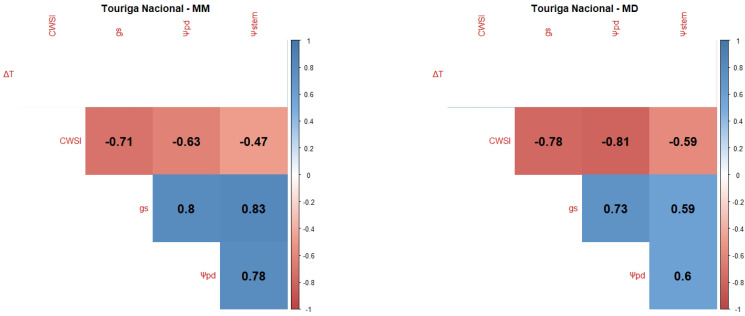
Correlograms showing Pearson Product–Moment Correlation Coefficients of the variety TN. Non-significant correlation coefficients (*p* > 0.05) are displayed in white with no value. Positive correlations are represented in blue and negative correlations are represented in red. The variety’s name is followed by MM for the correlation between MM measurements (**left side**) or MD for the correlation between mid-day measurements (**right side**).

Touriga Nacional (TN) presented strong positive correlations in the MM measurements between gs and $\Psi_{stem}$ and $\Psi_{pd}$ (Figure 4), while in the MD measurements, it presented negative correlations between the CWSI and $\Psi_{pd}$ and gs . On the other hand, it presented no correlations between ΔT and the other variables in the two measurements.

**Figure 5:**
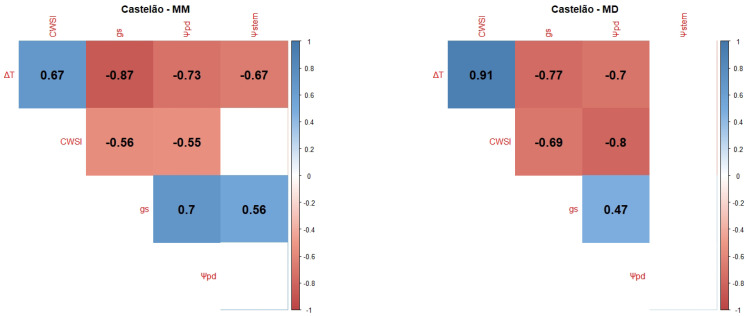
Correlograms showing Pearson Product–Moment Correlation Coefficients of the variety Cs. Non-significant correlation coefficients (*p* > 0.05) are displayed in white with no value. Positive correlations are represented in blue and negative correlations are represented in red. The variety’s name is followed by MM for the correlation between MM measurements (**left side**) or MD for the correlation between mid-day measurements (**right side**).

Castelão (Cs) presented strong negative correlations between ΔT and gs and $\Psi_{pd}$ in the MM measurements (Figure 5), and also a positive correlation between gs and $\Psi_{pd}$ . Unlike the other varieties, Cs’ strongest correlation was achieved in the MD measurements between ΔT and the CWSI (positive correlation), and another strong negative correlation between the CWSI and $\Psi_{pd}$ . During the MM, there were no correlations between $\Psi_{stem}$ and the CWSI and $\Psi_{pd}$ , and during the MD, there were no correlations between $\Psi_{stem}$ and all the other variables.

Generally speaking, all the varieties’ CWSI and ΔT present a positive correlation between themselves, and a negative correlation with the other WSIs. On the other hand, gs , $\Psi_{pd}$ , and $\Psi_{stem}$ tend to correlate positively between themselves, as expected.

### 2.2. Water Potential

**Figure 6:**
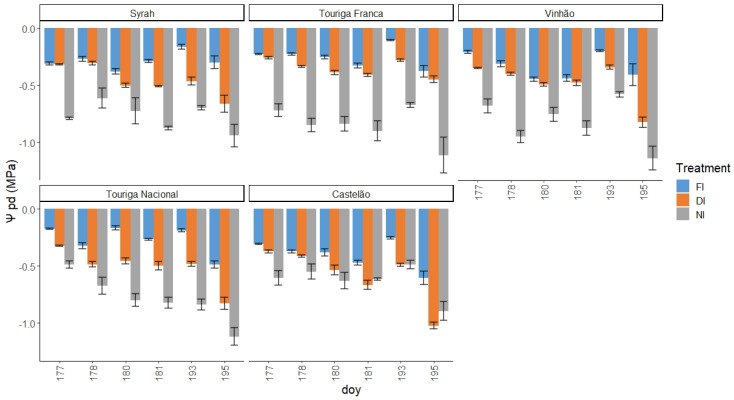
$\Psi_{pd}$ , in MPa, of the three treatments by variety, with standard errors, during the 6 days of measurements.

**Figure A1:**
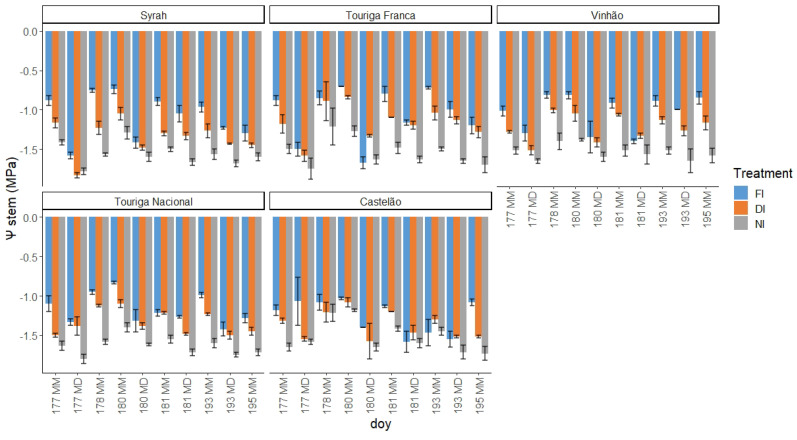
$\Psi_{stem}$ , in MPa, of the three treatments by variety, MM measurement and MD measurement, with standard errors, during the 6 days of measurements.

$\Psi_{pd}$ (Figure 6) and $\Psi_{stem}$ (Figure A1) at MM and MD during two water stress cycles were measured for full irrigation (FI), deficit irrigation (DI) (basal crop coefficient $K_{cb}=0.55$ ), and rainfed (NI) for the five grape varieties in the study. $\Psi_{pd}$ ranged from −0.17 MPa in FI to −1.14 MPa in the NI treatment. However, we should highlight that, for most of the varieties, the FI treatments presented a $\Psi_{pd}$ lower than expected according to threshold values for the absence of water stress (−0.3 MPa) [101].

$\Psi_{pd}$ showed significant differences between treatments in all varieties. Sy exhibited clear differences between NI and the other treatments, which were statistically similar. This behaviour lasted until DOY 181, when there was a clear division between all the treatments. On the last day (195), DI and NI were statistically similar. TF started like Sy, with FI and NI presenting significant differences, albeit small ones. After DOY 181, FI and DI started presenting significant differences, which remained until the final day. NI remained clearly different throughout the entire study. Vi started with a clear difference between NI and the other two treatments, which remained throughout the entire study, with the exception of DOY 195. TN behaved very differently from the other varieties, with each treatment being statistically different from the others throughout the entire study. This division between the treatments is completely apparent after DOY 178. Cs started with the three treatments being statistically different, despite an apparent similarity between FI and DI. This difference was absent the next day, with all three treatments being similar, a behaviour that would occur again on DOY 180. After this day, until the end of the study, DI and NI remained statistically similar, while FI maintained differences with the other two treatments (Figure 6).

Regarding $\Psi_{stem}$ , no significant differences were found between treatments for the two measurements (Figure A1), with the observed values ranging between −0.70 MPa (FI) and −1.83 MPa. In general, all varieties and treatments, even the well-watered ones, presented $\Psi_{stem}$ values that indicate a range of water deficits from moderate to severe and severe to high, according to the thresholds defined in [101] and the treatments presented.

### 2.3. Indicators of Crop Water Status Based on Canopy Temperature

Soil water deficits induce several types of plant responses over time. In particular, one of the most short-term physiological responses is stomatal closure, which reduces E and, consequently, evaporative cooling, resulting in an increase in leaf temperature.

#### 2.3.1. Normalised Canopy or Leaf Temperature with Reference to Air Temperature

**Figure A2:**
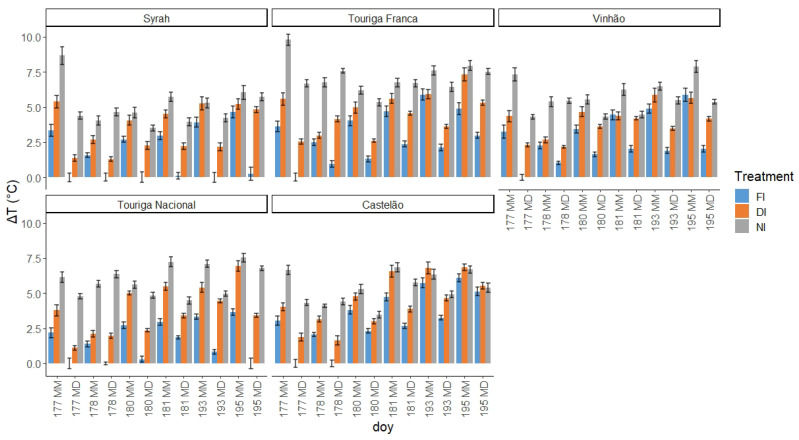
ΔT , in °C, of the three treatments by variety, MM measurement and MD measurement, with standard errors.

Sy and TN were the varieties in which the FI treatment was closer to the air temperature during the MD measurement. Sy maintained clear differences between the three treatments until DOY 193, where the DI and NI treatments were not significantly different. At DOY 195, the same behaviour was observed during the MM measurement, this time between all the varieties. This reverted in the MD measurement, where the FI temperature was very close to the air temperature. TF presented a higher ΔT in NI relative to the other varieties. Furthermore, on DOY 193 in the MM measurement, the FI and DI treatments had the same temperature. Vi displayed a big difference between NI and the other two treatments until DOY 180; on DOY 178, for example, FI and DI temperatures were virtually the same, while that of NI was much higher. After DOY 178, the three treatments’ temperatures were closer, except for FI in the MD measurement, until the last day, where there was a clear statistically significant difference between NI and the other two treatments. TN behaved similarly to TF, except for the last two days, where the discrepancies between NI and the other two treatments were higher. Cs presented clear differences between treatments until DOY 180. After this date, DI and NI treatments were almost always close to each other, and sometimes the same happened between FI and DI, e.g., on DOY 180 in the MD and DOY 193 in the MM measurements. On the last day, there were virtually no differences between the three treatments. The statistical significance of the irrigation treatment effect depends on the grape variety and measurement period (Figure A2).

#### 2.3.2. Crop Water Stress Index (CWSI)

**Figure A3:**
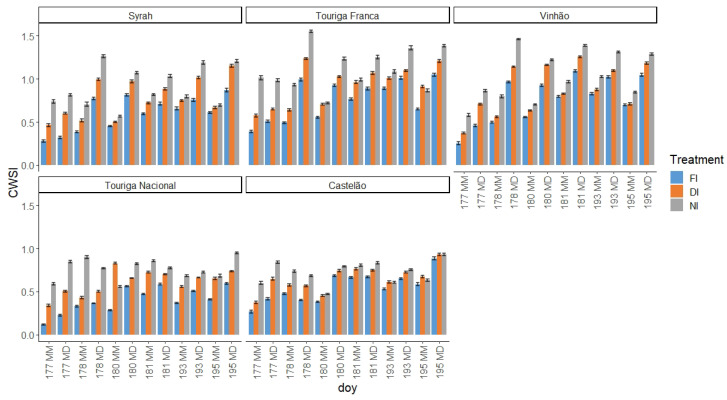
CWSI of the three treatments by variety, MM measurement and MD measurement, with standard errors.

Like ΔT , the CWSI is an indirect measurement of stress. The higher the value, the higher the stress. In this sense, DOY 178 was the day when the plants exhibited the most stress, especially TF, Vi, and, to a lesser extent, Sy. Looking at the other varieties, Cs was the variety that presented fewer differences between treatments, especially after DOY 180 (Figure A3). This index was clearly affected by the lower temperatures and VPD on DOY 180.

### 2.4. Stomatal Conductance

**Figure 7:**
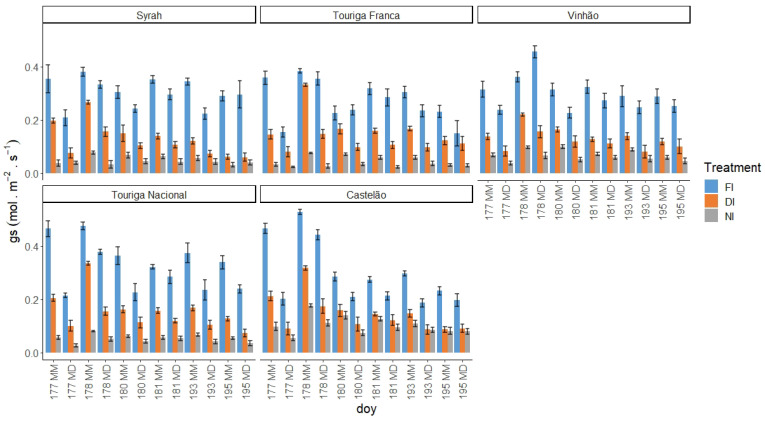
gs , in $\mathrm{mol}\;m^{-2}s^{-1}$ , of the three treatments by variety, MM measurement, and MD measurement, with standard errors.

gs was measured in two well-developed, sun-exposed leaves from 10 plants of each treatment and variety. As can be seen in Figure 7, the maximum gs occurred on DOY 178 in the morning, except for Vi, which exhibited the highest gs in the MD measurement. gs values ranged from 0.02 $\mathrm{mol}\;m^{-2}s^{-1}$ (TF, NI, MD) to 0.53 $\mathrm{mol}\;m^{-2}s^{-1}$ (Cs, FI, MM). During the first week of measurements, even though DOY 177 was the closest to the last irrigation event, the higher evaporative demand during that day (relative to the remaining days of that week) had a higher influence on all varieties and treatments, particularly in the MD measurement. These weather conditions affected stomatal behaviour, lowering the gs values. Generally, a daily decrease in gs was observed from MM to MD measurements, with the exception of Vi’s FI treatment on DOY 178. During the MM measurements, the only variety that always presented significant differences between all the treatments was TN. All the others showed similarities between DI and NI on DOY 195 (with the exception of TF, which displayed similarities between FI and DI on DOY 195), while Cs showed the same differences from DOY 180 to DOY 195. These behaviours extended to the MD measurement, with TN maintaining the differences between treatments and Vi behaving in the same way. During the first measurement cycle, gs exhibited lower values on DOY 177 (MD) in all grape varieties under study. This can be explained by the higher VPD (>6 KPa) and air temperature (40 °C). In contrast, no clear effects were observed due to the reduction in solar radiation that occurred on DOY 178 and 179, caused by the smoke cloud from the Canadian fires. Along the stress cycles, gs tends to present a negative slope, despite presenting no significant differences between treatments.

### 2.5. Models

With regard to the modelling aspect, we started by defining our objective: estimating $\Psi_{pd}$ . To achieve this, all models capable of solving regression problems in scikit-learn [102] were chosen. The Python library was tested against the dataset, with the default hyperparameters defined by the library. We ranked the results according to $R^{2}$ and Table 1 contains a representative selection of these results.

The three best-performing models, namely Gradient Boosting Regressor (GBR), ExtraTreesRegressor (ETR), and Random Forest Regressor (RFR), were selected for hyperparameter optimisation before final model evaluation. Optimisation was performed by maximising the coefficient of determination on the validation set with cross-validation. The highest-performing set of parameters was fixed, and the model was finally evaluated on the test set. The results of these steps can be found in Table 2.

Looking at the validation metrics, it is possible to observe that every single model improved, even if only by a couple of decimal places. GBR clearly had the biggest jump in performance, while ETR and RFR only exhibited a marginal improvement. In the end, the relative performance of these algorithms did not change. Looking at the difference between the validation and test scores, we can perceive that none of the regression models overfitted the available data.

**Figure 8:**
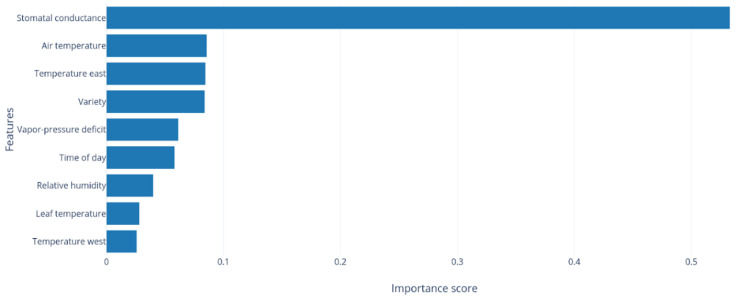
ExtraTrees Feature importance.

**Figure 9:**
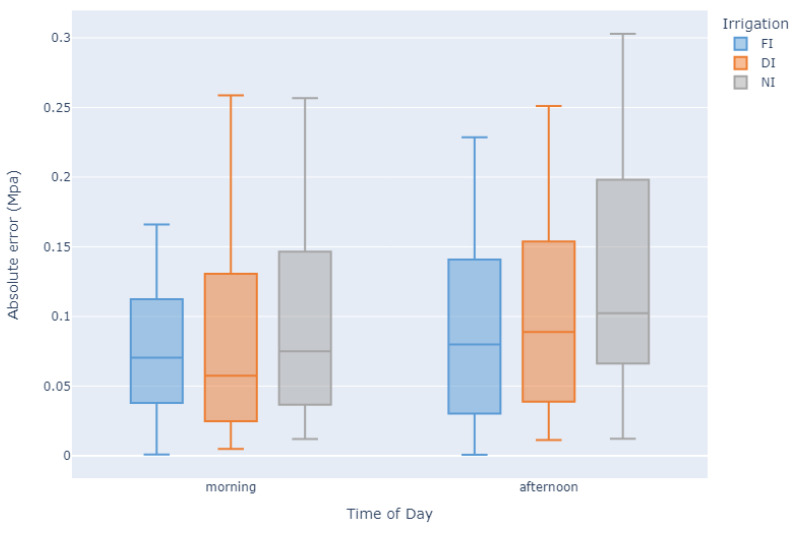
ExtraTrees absolute error for the different irrigation treatments and time of day when the field measurements were made.

In the end, the best result was an $R^{2}$ of 0.833 obtained by ETR with an MAE of 0.072. For further analysis of this algorithm, the feature importance was calculated according to the Gini importance method [103]. Looking at Figure 8, it is possible to observe that gs is by far the most important feature, followed by air temperature and canopy temperature from the east side. Moreover, by analysing each prediction’s absolute error by time of day and by irrigation regimen, as presented in Figure 9, we can observe which categories provide the best results, i.e., predictions for FI and for morning observations.

## 3. Discussion

One of the aims of this study consisted of the evaluation of direct and indirect indicators of grapevine water status on a sub-daily timescale as a tool for deficit irrigation management in grapevine vineyards. Although the presented data refer only to a single season, they are in accordance with studies in previous years [27,104] in the same region. The meteorological conditions observed during the experiment (average maximum temperature of 35.6 °C) were slightly higher than the normal climatological conditions of this region, characterised as ‘Csa’ under the Koppen–Geiger Climate Classification [105].

$\Psi_{leaf}$ can be considered the most straightforward indicator of a plant’s water status as it integrates the effects of soil, plant, and atmospheric conditions [106]. Typically, $\Psi_{pd}$ is considered as a reference method for grapevine WSIs since it is closely related to soil water potential in the root zone [101] and, consequently, as a method to ensure wine quality, since the grapevine water status directly influences the quality of grapes and wine [18,107,108]. $\Psi_{stem}$ is also considered a good indicator of the grapevine water status and an effective tool to differentiate between irrigation treatments [60,108]. However, our results showed no significant differences between irrigation treatments (Figure A1). This can be explained by the severity of meteorological conditions, like air temperature and VPD, in accordance with some authors [109,110], who state that, the closer to noon, the more difficult it is to discriminate between irrigation treatments. Our correlogram results (Figure 1, Figure 2, Figure 3, Figure 4 and Figure 5) support this statement, as the correlations between $\Psi_{stem}$ and the other WSIs are stronger during the MM measurements. Stronger MM correlations were also found between $\Psi_{pd}$ and gs . Rodrigues et al. [111] concluded that whenever VPD values are higher than 3 KPa, there should not be any differences between the $\Psi_{leaf}$ values of grapevines under different water stress conditions, once again showing the effect that a VPD can have on diminishing the differences between irrigation treatments. In fact, Suter et al. [106] stated that $\Psi_{stem}$ measurements are influenced by soil water availability and weather conditions, not allowing for temporal comparisons. This pattern appears to be what conditioned the usefulness of $\Psi_{stem}$ as a WSI in regions of elevated evaporative demand, as is the case in Reguengos de Monsaraz. With these data, we were able to conclude that, under our environmental conditions, $\Psi_{pd}$ is more effective than $\Psi_{stem}$ in determining the plants’ water status. Furthermore, the isohydric behaviour of some varieties can be exacerbated by these meteorological conditions. Differences in canopy leaf areas between varieties and treatments can explain some of the variations found in $\Psi_{pd}$ . In this sense, the reason why Cs’s water potentials are so similar between treatments may be related to its reduced canopy leaf area. Phenotypically less abundant than the others, Cs’s lower canopy area, and consequently reduced E area, diminish soil water depletion [108].

Thermal indices have been widely used to estimate crop water status. Among the many indices, the most used are the CWSI [112,113] and ΔT [114], which have been recently used in viticulture (remote and proximal sensing) [115]. Sy and TN are the two varieties that presented the lowest ΔT regarding the FI treatment. Corroborating this, TN is the variety that presented the highest differences in ΔT between FI and NI treatments, behaving like an isohydric variety, in agreement with Blanco-Ward et al. [116] and in disagreement with Lovisolo et al. [117] and Costa et al. [118]. This behaviour is directly correlated with the gs variation. In fact, this variety seems to present anisohydric behaviour when fully irrigated, and isohydric behaviour under water stress conditions, contributing to explaining the contrasting behaviours found in the literature [116,117,118]. Cs and TF, on the other hand, presented higher ΔT values, particularly during the second water stress cycle, where the meteorological conditions were relatively stable. Regarding the CWSI, Cs and TN presented the lowest values. However, the two varieties were differently affected by the irrigation treatments—Cs presented virtually no differences between treatments, contrary to what was observed for TN. Even though the suggestion of García-Tejero et al. [119] that the CWSI may be a more robust indicator of water stress, DOY 180 at MM is proof of the meteorological influence on the thermal indices’ ability to discriminate between treatments, reducing the thermal indices’ effectiveness as a WSI estimator.

gs is also a robust variable indicator of the plants’ water status [108]. According to Patakas et al. [120], gs and the photosynthetic rate were significantly lower under water stress conditions. In fact, all the varieties presented a high level of correlation between gs and the thermal indices, with the exception of the correlation between ΔT and gs at MD in TN, and between the CWSI and gs in Vi. The strong correlation between gs and ΔT is similar to what was found by Baluja et al. [82], and the correlation with the CWSI may mean, as Costa et al. [118] concluded, that the leaf temperature and the CWSI are linked with gs . Therefore, it is important to calculate more than one index in order to obtain a more robust dataset. It is, however, important to note that there are several factors influencing the canopy temperature obtained by thermal cameras besides the more frequently accounted for (air temperature and relative humidity), such as wind speed, solar radiation intensity, and canopy features, namely leaf size or canopy porosity [121].

Regarding the correlations between gs and $\Psi_{pd}$ , a stronger link was observed between these two WSIs during the MM measurements. Such a pattern indicates an influence of the more severe weather conditions during the MD measurements on diminishing gs differences between irrigation treatments. Cs’s gs behaviour under NI follows that of its $\Psi_{pd}$ and is once again linked to the lower canopy area [108]. Despite Sy’s $\Psi_{pd}$ under FI and DI at DOY 177 and 178 presenting no significant differences, gs did not follow this behaviour, as it was statistically different, suggesting a putative effect of the cumulative water stress.

According to García-Tejero et al. [119], the best time to monitor vine’s water status (namely gs and photosynthetic rate) is between 2 and 5 pm, the time of the day when there are more differences in temperature between different irrigation treatments. Our varieties’ behaviour contradicts this finding, especially taking into consideration the meteorological conditions (high air temperature and VPD, especially on DOY 177). Cs, however, did not exhibit such differences, which may be explained, once again, by its stable gs and more anisohydric behaviour. The best correlations can be found in the morning (Figure A2), when the air temperature and VPD were not as harsh as in the MD measurement. This indicates that temperature and relative humidity have an effect on the physiological parameters of the grapevine, which can mask, or make less evident, the effect of soil water depletion. This conclusion refutes the findings of Williams et al. [122], who found a strong correlation between MD measurements of $\Psi_{leaf}$ and gs with daily water use in grapevines. Even so, this effect is evident on gs with similar FI and DI conditions during the morning. With differences in the soil water status, if the atmospheric conditions are not limiting, Sy and TF tend to maintain a high E. This behaviour is less evident in Vi.

Taking into account that the best correlations were generally found during the MM period, namely between $\Psi_{pd}$ and gs , it must be noted that ΔT and the CWSI presented better correlations with $\Psi_{pd}$ during the MD ( ΔT in Sy, TF, and Vi, CWSI in TN and Cs), contrasting with the behaviour of gs . However, some discrepancies were found for TN, where no significant correlations were found for ΔT . In this case, the CWSI showed a better correlation with the other variables. Vi, on the other hand, showed a better correlation with ΔT , without any correlation with the CWSI. One possible explanation is the contrasting canopy architecture between Vi and TN. Vi usually presents a more erect and less dense canopy, while TN usually presents a more recumbent and denser canopy. These features may influence the temperature dissipation patterns of each variety, therefore affecting each thermal index, as was stated in [121]. The thermal correlations evidence the importance of performing each thermal WSI measurement at different periods of the day.

In general, the $\Psi_{stem}$ measured at MD showed no significant correlation with the other variables, with the exception of TN. For all varieties and measurement periods, gs always presented significant correlations with $\Psi_{pd}$ , proving to be the most reliable WSI for estimating $\Psi_{pd}$ .

Regarding the modelling of grapevine water potential, although some works may not be directly comparable—given the differences in predictors, target variables, platform used, varieties, and methods of model evaluation—in Table 3, we provide an overview of the results in the literature.

A comparison with the literature shows that our results are on par with the state of the art, even when compared against studies involving hyperspectral spectroscopy, which usually provides a more refined set of spectral information about the plant and therefore achieves higher evaluation scores. With regard to the models’ performance and model selection following our multi-model testing approach, the subset of best-performing models falls into the category of ensemble learning methods, which have been referenced in the literature as being robust for agricultural data modelling [124,125]. In Figure 9, a box plot of the absolute error for each model prediction on the training and test sets shows that, generally, observations that were obtained in the MM period are more relevant to the modelling of $\Psi_{pd}$ , i.e., the errors are lower. For a practical application of this technique, only a measurement during the morning should be needed. This will not only give more accurate results, but also reduce the effort needed for data collection. The results show that by only using the morning observations, the mean average error is 0.083 MPa, while when only using the MD observations, we can expect a mean average error of 0.105 MPa. Both of these values are still within an acceptable margin. In relation to the impact of water stress on the predictions, the less water-stressed the plant is, the more accurate the prediction will be. Looking at the mean average error for each of the irrigation treatments, NI has the highest value at 0.109 MPa, followed by DI with 0.93 MPa and finally FI with the lowest error at 0.080 MPa. While the error does indeed increase, it is still within an acceptable margin, so even a winegrower who is following a DI approach can use our model to accurately estimate $\Psi_{pd}$ for irrigation management.

## 4. Materials and Methods

### 4.1. Plant Material and Experimental Site

The study was conducted during the 2023 growing season at the Ampelographic Field of Herdade do Esporão (CAHE) located in Reguengos de Monsaraz, Alentejo, Portugal (38.380098∘ N, 7.560724∘ W). The region has a temperate climate with hot and dry summers, characterised as ‘Csa’ under the Köppen–Geiger Climate Classification [105]. The historical records from WorldClim 2.1 [126] show an average annual temperature of 16.1 °C, while the annual precipitation is 572 mm. The soil in this area is characterised as a Eutric Cambisol with an ApBw1Bw2C profile; it is granite-derived and consists of 75–80% sand content, with a pH ranging from 7.0 to 7.6, a low organic matter content, and a high content of phosphorus and potassium.

The vineyard is composed of 189 rows, and in each row, a single different variety is planted. The grapevine plants, aged twelve years, were grafted onto 1103P rootstocks and were spaced at intervals of 3 m between north–south oriented rows and 1.5 m between plants. The training system used was a vertical shoot positioned system, spur-pruned on a bilateral Royat cordon system. All vines were pruned evenly, with 16 buds per vine.

This study involved five grapevine varieties: Sy, TF, Vi, TN, and Cs. Sy is considered as anisohydric by some authors [54,127], but presented a near-isohydric behaviour in a study by Pou et al. [56]. TN is considered by some authors as anisohydric [117,118], while others consider TN as isohydric [116]. Vi was studied by Barreales [128], whose findings point to a near-isohydric tendency, a result also observed by Damásio [104], while Cs was studied by Santos et al. [129], whose results point to anisohydric behaviour, a result also observed by Damásio [104]. There is a lack of information regarding TF’s behaviour; however, Jacinto et al. [27] studied 172 varieties, TF included, and grouped it in a middle cluster according to the carbon-13 enrichment measured in berries and phloem, indicating a near-isohydric behaviour. These varieties were selected based on their contrasting carbon-13 signature results [27] and their significance within the Alentejo Wine Region.

All varieties have been subjected to three different irrigation treatments since 2018: FI (100% crop evapotranspiration ( $ET_{c}$ )); DI (50% of FI); and NI. The FI treatment involved weekly irrigation (*I*), calculated as $I=ET_{0}\times K_{cb}\times 1.1$ , where the reference ET ( $ET_{0}$ ) represents the daily ET calculated using the Penman–Monteith FAO 56 method [130] and $K_{cb}$ is the estimated crop coefficient derived from the formula $K_{cb}=1.44\times \mathrm{NDVI}-0.1$ [39]. The use of the factor of 1.1 accounts for soil water evaporation. On the other hand, the NI treatment relied solely on rain.

For the present study, 10 homogeneous plants per irrigation treatment were randomly selected for each variety, taking into account the vigour and health. From these 10 plants, 5 were chosen for the Ψ measurements.

The study was performed over two weeks: week 1, with measurements on DOY 177, 178, 180, and 181; week 2, with measurements on DOY 193 and 195. The plants under FI and DI treatments were irrigated at the end of the day on DOY 174 (12.2 and 6.1 millimetres (mm))—3 days before the beginning of the study—DOY 182 (13.6 and 6.8 mm), DOY 185 (8.0 and 4.0 mm), DOY 188 (8.0 and 4.0 mm)—5 days before the start of the second week—and DOY 195 (7.8 and 3.9 mm). These DOYs were chosen to coincide with the beginning of the maturation period, a stage where a more intense deficit irrigation is usually applied for grape quality reasons [131]. The irrigation depth corresponds to the FI and DI treatments, respectively. These irrigation depths were defined in accordance with the farmer’s irrigation scheduling. The NI treatment was not irrigated, and no precipitation was recorded during the weeks prior to the study. In order to obtain the maximum possible differences between irrigation treatments, it was decided that the first cycle of measurements would start 3 days after the last irrigation. The irrigation cycle was resumed the day after the first week of measurements, in a weekly schedule, until 5 days before the beginning of the second week of measurements in order to observe a higher influence of soil water depletion, which we expected to exacerbate the differences between DI and FI treatments.

### 4.2. Meteorological Monitoring and Assessment

**Figure 10:**
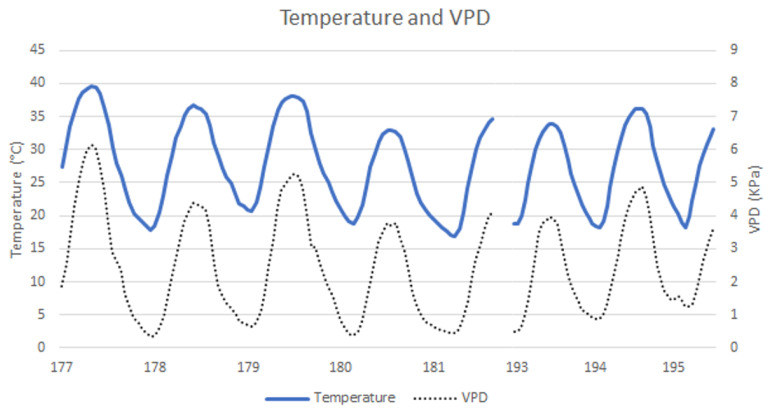
Temperature and VPD during the study.

Temperature and relative humidity data were collected with a CS215 temperature and relative humidity probe (Campbell Scientific, Inc., Logan, UT, USA). These measurements were recorded within a 1 min interval using a Campbell CR1000 datalogger (Campbell Scientific, Inc., Logan, UT, USA). The VPD was then calculated using the obtained temperature and relative humidity data (Figure 10).

### 4.3. Grapevine Water Status

The grapevine water status was monitored through the measurement of $\Psi_{pd}$ , and through $\Psi_{stem}$ measurements at 10:30 a.m. and 1:30 p.m. using a Scholander pressure chamber (Manofrigido, S.A., Lisbon, Portugal). In each sampling, measurements were taken from five plants per variety and treatment, using at least 2 leaves per vine.

### 4.4. Stomatal Conductance

gs measurements were performed with a steady-state porometer (LI-680, LI-COR Inc., Lincoln, NE, USA). Measurements were taken at 10:30 a.m. and 1:30 p.m. in 2 leaves per vine from 10 plants per variety and treatment.

### 4.5. In Situ Thermal Imagery

Thermal imagery measurements were taken with a thermal camera (FLIR-C5, Teledyne FLIR LLC, Wilson Ville, OR, USA). Measurements were taken at 10:30 a.m. and 1:30 p.m. in 10 plants per variety and treatment, and the images were taken from both the east and west sides of the canopy. The camera was placed at 1 m distance from the monitored plants. The following thermal indices were calculated according to Jones [132]: ΔT (1) and the crop water stress index (CWSI) (2)

(1) $$\Delta T=T_{c}-T_{air}$$

(2) $$CWSI=\frac{T_{c}-T_{wet}}{T_{dry}-T_{wet}}$$

### 4.6. Statistical Analysis

The experimental design consisted of a single factor analysis for each variety, for which the factor was the irrigation treatment, with three levels: FI, DI, and NI. For each variety, a one-way ANOVA was performed. The differences between each WSI between treatments were calculated for each variety. FI to DI, FI to NI, and DI to NI differences were tested through a nonparametric Kruskal–Wallis test for a significance level of α = 0.05. Correlogram graphs were built using *Hmisc* and *corrplot* R packages.

All statistical analyses and plotting were performed using R Statistical software version 4.3.1 (R Core Team, 2022).

### 4.7. Machine Learning Methodology

In our modelling approach, we considered $\Psi_{pd}$ to be the target variable and used meteorological variables, gs , and thermal data as predictor variables. To model $\Psi_{pd}$ , we tested multiple machine learning regression algorithms. Due to the large number of models tested, the evaluation process was conducted in three steps (detailed in Section 4.7.3).

#### 4.7.1. Dataset Creation

The creation of a dataset for the machine learning models involved gathering the following information:

- Plant description (grape variety, irrigation treatment);
- Meteorological information (relative humidity, air temperature);
- Thermal images;
-
gs records;
-
$\Psi_{pd}$ records.

The thermal images were manually analysed using FLIR Thermal Studio to extract the canopy temperature from both sides of the vine. This analysis resulted in a table with one line per plant per day per time of day, with a record of the average canopy temperature for both sides of the vine.

The gs records (porometer) and meteorological information (automatic weather station) were already in a tabular format, so it was only a matter of filtering the porometer readings to consider solely the vines where $\Psi_{pd}$ was collected and then matching the time of these records with the time label of each weather station data entry.

The final dataset contained data from the 2023 season gathered over 6 days (177, 178, 180, 181, 193, and 195), with 2 measurements per day (MM and MD), 5 varieties (Sy, TF, Vi, TN, and Cs), 3 irrigation treatments (FI, DI, and NI), and finally 5 measurements on different vines, resulting in a total of 900 data points. The features of each data point are listed in the “variable” column in Table 4.

We followed the approach used by Fernández-Novales et al. [88] and did not include the CWSI calculation in our dataset to avoid having to deal with reference temperatures, which are still a practical impediment when defining thermography-based automated methods, but included meteorological data from a nearby station to support the modelling of the crop environment system.

A stratified random split [133] was applied to the dataset, with 75% for training and 25% for testing. The stratification took into account the following variables: variety, time of measurement, and irrigation treatment. This means that the test set and the training set had approximately the same distribution of the defined strata, making it a more representative sample of the collected data.

#### 4.7.2. Models

Regarding the modelling approach, as previously stated, the objective was to predict $\Psi_{pd}$ ; this meant that we were facing a supervised regression problem. This being the case, all the models in the scikit-learn (1.3.0) library [102] capable of regression were tested, giving a broad idea of what off-the-shelf models were better suited to predict $\Psi_{pd}$ . This approach did not assume any prior bias towards the performance of any specific model, providing an equal starting point for all models. The tested models ranged from simple linear models, support vector machines (SVMs), and artificial neural networks (ANNs) up to ensemble models.

The top 3 models were ensemble learning methods (consisting of two bagging methods (ExtraTreesRegressor, RandomForestRegressor) and one boosting method (GradientBoostingRegressor)) that, according to Hastie et al. [134], can be described by the following:

*The idea of ensemble learning is to build a prediction model by combining the strengths of a collection of simpler base models. (…) Ensemble learning can be broken down into two tasks: developing a population of base learners from the training data and then combining them to form the composite predictor.*

Several combinations of data pre-processing methods were also tested: min–max scaling, where each feature is rescaled in order to make the minimum value 0 and the maximum value 1; principal component analysis (PCA) for dimensionality reduction; a standard scaler, where the data are rescaled to have mean 0 and standard deviation 1; and finally, normalisation, where each row is scaled to add up to one [135].

#### 4.7.3. Evaluation Methodology

Regarding the model evaluation methodology, the primary indicator for evaluating the models was the coefficient of determination ( $R^{2}$ ) (Equation (3)) since it is a good metric for regression tasks. The MAE (Equation (4)) was also calculated, but only to give an idea of the expected error of the model in the same units as the $\Psi_{pd}$ .

(3) $$R^{2}=1-\frac{\sum_{i=1}^{n}{\left(y_{i}-{\hat{y}}_{i}\right)}^{2}}{\sum_{i=1}^{n}{\left(y_{i}-{\bar{y}}_{i}\right)}^{2}}$$

(4) $$MAE=\frac{1}{n}\sum_{i=1}^{n}\left|y_{i}-{\hat{y}}_{i}\right|$$

where *n* is the number of samples in the test set, $y_{i}$ is the true value for sample *i*, $\hat{y}$ is the predicted value of *y* for the *i*-th sample, and $\bar{y}$ is the mean value of *y*.

The evaluation process was divided into three steps:

1. Model ranking;
2. Hyperparameter optimisation;
3. Final model evaluation.

In the model ranking step, all of the models were tested with the default hyperparameters defined by the scikit-learn (1.3.0) library. This ranking was determined only on the training set using leave-one-group-out cross-validation [136] in order to make the most out of the low amount of data. This means that groups were defined according to grape variety, irrigation treatment, and date of observation. Subsequently, leave-one-group-out was used to evaluate the model and train the model on the remaining k−1 groups. This process was repeated by every group until all groups had taken their turn being left out for evaluation. In the end, *k* similar model instances were evaluated on different data, and the evaluation metrics were averaged [137]. Once this step was concluded, the models were ordered according to the $R^{2}$ and the top three were selected for the next step.

During the hyperparameter optimisation step, the main objective was to find the set of hyperparameter values that maximises the $R^{2}$ score of the selected models. Once again, in this step, leave-one-group-out cross-validation was performed on the training set in order to evaluate the hyperparameterisations. For a detailed list of the optimised hyperparameters and the range of the search, see Appendix B.

Having found the best models and optimised their hyperparameters, the final model evaluation was performed with these models and their respective hyperparameterisation over the test set. The test set was only used in this final step to obtain a credible performance estimate of the models with never-before-seen data. For the training procedure, the full training set was used.

**Figure 11:**
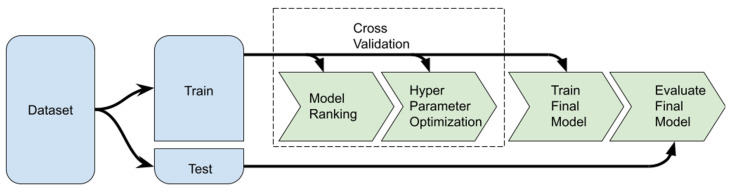
Diagram of the machine learning methodology.

In short, the models were evaluated with default hyperparameters on the training set using cross-validation to obtain a preliminary rank of all the available models. This ranking gave a general idea of the best models for this dataset. The next step involved using hyperparameter optimisation on the top three models, once again only using the training set, with the purpose of achieving further performance gains. Once all three models had been optimised, they were once again trained, but this time using the full training set and only then evaluated on the test set. A full diagram of the testing methodology can be seen in Figure 11.

## 5. Conclusions

With this work, we intended to (i) evaluate which of the two Ψ methods ( $\Psi_{pd}$ or $\Psi_{stem}$ ) is the more robust for determining the plant water status in an arid region with high evaporative demand, (ii) identify the variables that demonstrate superior predictive capabilities in forecasting the more robust Ψ , and (iii) model the Ψ selected in point (i).

Concerning the WSIs, $\Psi_{stem}$ , in particular that measured at MD, proved, under our test conditions, not to be a reliable WSI, and this can be attributed to the additional effect on stomatal behaviour caused by the high VPD and air temperature. On the contrary, $\Psi_{pd}$ showed significant differences between treatments in all varieties, validating the choice of this variable as the target for WSI modelling with machine learning. The result of the machine learning modelling approach presented an $R^{2}$ of 0.833 for the ExtraTrees ensemble method. Of all the variables used in the modelling, the one with the highest relevance was gs , which confirmed the correlations obtained between gs and the other WSIs. In this work, we present an approach for the reliable and automated estimation of $\Psi_{pd}$ in a non-invasive and quick way, making it possible to estimate values without depending on heavy and specialised equipment, such as a Scholander chamber, and without the need to collect reference canopy temperature values, making it an important step towards an automated vineyard irrigation management system. Climate change brings challenges to viticulture, particularly in Mediterranean-like regions, increasing the necessity for irrigation due to higher temperatures and VPDs, along with water scarcity. This reality brings forth the need for better irrigation management. Understanding and estimating the plants’ water status help farmers to adjust irrigation scheduling in a more precise way. In this sense, the model presented in this work can be incorporated into agricultural management platforms, helping farmers to calculate the crop irrigation requirements, schedule irrigation, and optimise the irrigation depth. 

## Figures and Tables

**Table 1:** A selection of the cross-validation performances of the models with default parametrisation. “Rank” is the number of the relative position of the models sorted according to the coefficient of determination ( $R^{2}$ ), “Model” is the model’s name in the scikit-learn library, “Module” is the name of the module where it is located in the scikit-learn library, a good indication of the family of algorithms it belongs to, and the mean absolute error (MAE) is also displayed.

Rank	Model	Module	$R^{2}$	MAE (MPa)
1	GradientBoostingRegressor	Ensemble	0.761	0.097
2	ExtraTreesRegressor	Ensemble	0.756	0.101
3	RandomForestRegressor	Ensemble	0.709	0.107
6	SVR	SVM	0.687	0.113
7	NuSVR	SVM	0.676	0.118
9	LassoLarsIC	Linear	0.599	0.132
10	LinearRegression	Linear	0.596	0.132
25	DecisionTreeRegressor	Tree	0.548	0.130
29	MLPRegressor	Neural Network	0.490	0.140
31	KNeighborsRegressor	Neighbors	0.445	0.155
36	GaussianProcessRegressor	Gaussian Process	0.218	0.146
42	RadiusNeighborsRegressor	Neighbors	−0.029	0.213

**Table 2:** The validation and test metrics of the top three algorithms after hyperparameter optimisation.

Model	Validation		Test	
	$R^{2}$	**MAE (MPa)**	$R^{2}$	**MAE (MPa)**
GradientBoostingRegressor	0.786	0.093	0.830	0.073
ExtraTreesRegressor	0.759	0.100	0.833	0.072
RandomForestRegressor	0.710	0.107	0.798	0.079

**Table 3:** Literature comparison of results sorted by $R^{2}$ , except for our approach that is positioned in the first row.

Reference	No. of Varieties	Target	Predictors	Model	Platform	$R^{2}$
**Our approach**	**5**	$\Psi_{\mathrm{pd}}$	**Thermal imaging, gs, meteorology**	**ExtraTrees**	**Handheld**	**0.83**
[96]	2	$\Psi_{pd}$	Hyperspectral bands	Algorithm based on the search for covariance	Handheld	0.97
[84]	1	$\Psi_{stem}$ (MD)	Hyperspectral bands	Artificial Neural Network	UAV	0.87
[90]	6	$\Psi_{stem}$ (MD)	NIR spectrometer	Rotation Forest, M5 trees	Handheld	0.84
[97]	1	$\Psi_{leaf}$ (MD)	CWSI	Linear Regression	UAV, Field sensors	0.83
[92]	1	$\Psi_{stem}$ (MD)	NIR spectrometer	Partial Least Squares	ATV	0.69
[98]	1	$\Psi_{leaf}$ (MD)	CWSI	Linear Regression	UAV	0.51
[123]	2	$\Psi_{pd}$	Thermal indices	Two-way Analysis of Variance (ANOVA)	UAV, Handheld	0.50

**Table 4:** A list of the inputs to the model and their respective source.

Source	Variable
Porometer	gs Leaf temperature
Thermal Camera	Temperature canopy east Temperature canopy west
Manually Recorded	Time of day (MM/MD) Variety
Meteorological Station	VPD Relative humidity Air temperature

## Data Availability

Due to the nature of the research, due to commercial restrictions, the supporting data are not available.

## Appendix A

This appendix presents graphs for the WSIs measured during this study: $\Psi_{stem}$ (Figure A1), ΔT (Figure A2), and the CWSI (Figure A3) at MM and MD, during the two water stress cycles for FI, DI, and NI and for the five grape varieties studied.

## Appendix B

This appendix contains the definition of the search space for the top 3 models after the model ranking step. When presented with [a, …, b] it means that for that parameter, the search space ranged from a minimum of a to a maximum of b. When no ellipses are present, this means that all options are listed inside the square brackets.

**Table A1:** The search space for the hyperparameter optimisation for GradientBoostingRegressor along with the selected optimal values.

Hyperparameter	Search Space	Optimal Value
n_estimators	[50, …, 1000]	585
min_samples_split	[2, …, 133]	6
min_samples_leaf	[1, …, 133]	6
loss	[squared_error, absolute_error, quantile]	squared_error
learning_rate	[1 × 10^−5^, …, 100]	0.020596
subsample	[0.2, …, 1]	0.4
max_depth	[1, …, 1000]	75

**Table A2:** The search space for the hyperparameter optimisation for ExtraTreesRegressor with the selected optimal values.

Hyperparameter	Search Space	Optimal Value
n_estimators	[50, …, 1000]	528
min_samples_split	[2, …, 133]	2
min_samples_leaf	[1, …, 133]	1
bootstrap	[False, True]	False
criterion	[squared_error, friedman_mse]	squared_error

**Table A3:** The search space for the hyperparameter optimisation for RandomForestRegressor with the selected optimal values.

Hyperparameter	Search Space	Optimal Value
n_estimators	[50, …, 1000]	806
min_samples_split	[2, …, 133]	2
min_samples_leaf	[1, …, 133]	1
bootstrap	[False, True]	True
criterion	[squared_error, friedman_mse]	squared_error
