# Correction: Damásio et al. Can Grapevine Leaf Water Potential Be Modelled from Physiological and Meteorological Variables? A Machine Learning Approach. *Plants* 2023, *12*, 4142

**DOI:** 10.3390/plants15121764

**Published:** 2026-06-08

**Authors:** Miguel Damásio, Miguel Barbosa, João Deus, Eduardo Fernandes, André Leitão, Luís Albino, Filipe Fonseca, José Silvestre

**Affiliations:** 1 INIAV I.P., Instituto Nacional de Investigação Agrária e Veterinária, Polo de Inovação de Dois Portos, Quinta da Almoinha, 2565-191 Dois Portos, Portugal; joao.deus@iniav.pt (J.D.); jose.silvestre@iniav.pt (J.S.); 2 SISCOG SA, Sistemas Cognitivos, Campo Grande, 378 - 3°, 1700-097 Lisboa, Portugal; miguel.barbosa@siscog.pt (M.B.); eduardo.fernandes@siscog.pt (E.F.); andre.leitao@siscog.pt (A.L.); luis.albino@siscog.pt (L.A.); filipe.fonseca@siscog.pt (F.F.); 3 GREEN-IT Bioresources4sustainability, ITQB NOVA, Av. da República, 2780-157 Oeiras, Portugal; 4 GI-1716 Projects and Planification, Agroforestry Engineering Department, Escuela Politécnica Superior de Ingeniería Lugo, University of Santiago de Compostela, 27002 Lugo, Spain

## Additional Affiliation

In the published publication [1], there was an error regarding the affiliation for Miguel Damásio. In addition to affiliation 1, the updated affiliation should include GI-1716 Projects and Planification, Agroforestry Engineering Department, Escuela Politécnica Superior de Ingeniería Lugo, University of Santiago de Compostela, 27002 Lugo, Spain. The authors state that the scientific conclusions are unaffected. This correction was approved by the Academic Editor. The original publication has also been updated.
